# Longitudinal Improvements in Zoo-Housed Elephant Welfare: A Case Study at ZSL Whipsnade Zoo

**DOI:** 10.3390/ani10112029

**Published:** 2020-11-04

**Authors:** Katherine Finch, Fiona Sach, Malcolm Fitzpatrick, Nic Masters, Lewis J. Rowden

**Affiliations:** Zoological Society of London, Outer Circle, Regent’s Park, London NW1 4RY, UK; k.finch@chesterzoo.org (K.F.); fiona.sach@zsl.org (F.S.); malcolm.fitzpatrick@zsl.org (M.F.); nic.masters@zsl.org (N.M.)

**Keywords:** animal welfare, Asian elephant, *Elephas maximus*, SSSMZP, behaviour, social, resting

## Abstract

**Simple Summary:**

Zoo elephant welfare has been the topic of much debate over the last two decades, with criticisms made regarding the husbandry and welfare of these species held in European and North American zoos. The aim of this study was to evidence the value of a species-specific behavioural monitoring programme and highlight the positive improvements in elephant welfare that were made in a single collection case study, by the comparison of behavioural activity budgets (time spent performing a particular behaviour) with previous published literature. This study identifies numerous indicators of positive welfare in our collection, including species-appropriate levels of feeding, low engagement in stereotypy (abnormal repetitive behaviour), and proportions of resting behaviour that are consistent with figures published from comparative zoo individuals. Additionally, we show that positive social associations exist between individuals in our study group, with low incidences of agonistic social behaviour and high engagement in positive social interactions. Finally, we acknowledge that improvements are required to further enhance elephant welfare in zoos and we have used the data collected throughout this research programme to adopt an evidence-based approach to the husbandry and management of Asian elephants at Zoological Society of London (ZSL) Whipsnade Zoo.

**Abstract:**

Over the last two decades, criticisms were raised regarding the welfare experienced by elephants in European and North American zoos. Concerns regarding the welfare of zoo-housed elephants in the UK and Europe were consolidated in the publication of several key reports, and media interest peaked. Throughout this study we aim to outline the behavioural measures of welfare observed in the current group of Asian elephants (*Elephas maximus*) at Zoological Society of London (ZSL) Whipsnade Zoo, using key welfare indicators for this species and comparing them to previous published work. Following the instigation of a species-specific research programme, empirical behavioural data were available to quantify any developments in care and welfare. The collection of behavioural information revealed that individuals in our study group engage in low levels of stereotypic behaviour, have formed and maintain strong associations with one another and display a high proportion of engagement in lying rest. We outline that by applying simple, low-cost methods of behavioural data collection and analysis, it is possible to collect evidence that allows us to evaluate individual level welfare. This facilitates the adoption of an evidence-based approach to zoo management as well as demonstrating compliance with updated legislation for this species.

## 1. Introduction

There are multiple roles which a 21st century zoological collection strives to fulfil. Institutions are now expected to not only provide an engaging visitor experience [1], but also to deliver conservation related education programmes [2] and conduct scientific research [3]. However, the provision of optimal animal welfare and husbandry practices is widely acknowledged to be the primary goal of any modern zoo [4,5,6,7].

Animal welfare can be described as the state of an individual in relation to its environment [8] and requires a multidimensional approach to assessment [9]. The value of using behavioural data in assessing welfare is increasingly highlighted. Behavioural measures are non-invasive, cheap to measure and can be easily repeated over time [10]. Multiple studies outline unique behavioural measures of welfare for zoo species, which can include both positive and negative indicators [11,12,13]. The importance of optimising animal welfare cannot be understated, with individuals experiencing a positive welfare state suggested to be best placed to contribute meaningfully towards conservation breeding programmes [14], the most behaviourally competent [15] and most likely to enhance visitor experiences [16].

To ensure that animals experience a high level of welfare in a zoo setting, there is specific guidance and legislation that all zoos must adhere to, some of which is specific to certain species or taxonomic groups [17]. British zoos are required to meet the animal welfare standards set out in the Zoo Licensing Act of 1981 [18]. The Secretary of State Standards of Moderns Zoo Practice (SSSMZP) [19] provide the necessary guidance to zoo operators and inspectors on the Act’s implementation and set out the minimum standards that zoos are expected to meet [20,21].

Over the last two decades, elephant welfare within zoos across Europe has been the topic of much investigation following publication of studies which criticised the standard of care elephants receive in zoo environments [22,23]. As with other large, highly intelligent and socially complex species with a vast natural home range, there are concerns that it is not possible to meet the basic needs of elephants in captivity [24]. Many of these concerns for captive elephants housed specifically in the United Kingdom (UK) were documented in a report published in 2008 by Harris et al. [23]. This report included data on 77 elephants (both Asian *Elephas maximus* and African *Loxodonta africana*) managed in 13 UK zoos at the time. The age ranges of these individuals were from 0.6–50 years for Asian elephants and 0.5–40 years for African elephants. The main findings of this report outlined a high engagement in abnormal repetitive behaviours, poor social compatibility between individuals and concerns with obesity, all issues suggested to be associated with poor zoo management practices at the time or historically.

As a response to the findings of the report by Harris et al. [23], the UK Government raised concern about the keeping of elephants in UK zoos and invited the Zoos Forum Report (now the Zoo Expert Committee) to review the situation. They recommended the establishment of an Elephant Welfare Group (EWG), designed to demonstrate evidence of improved welfare of UK zoo elephants over a 10-year period. The UK Government followed this recommendation and tasked the British and Irish Association of Zoos and Aquariums (BIAZA—previously known as the Zoo Federation) to coordinate the work. The EWG is a multi-stakeholder group that has since worked together to develop monitoring tools and protocols for the consistent assessment of body condition, locomotion, foot health and overall welfare in elephants [25]. Frameworks for assessing captive elephant welfare currently include the UK Elephant Behaviour Welfare Assessment Tool and Health Pack which all institutions are required to complete quarterly [12]. In addition to this, as part of their zoo license, all UK zoos holding elephants are inspected by Department for Environment, Food and Rural Affairs (DEFRA) appointed inspectors, against Appendix 8 of the SSSMZP—An elephant specific legislative guidance document. Appendix 8 was updated in 2017 to align elephant management standards with current best practice for the species, with the aim to enhance elephant welfare [26]. On a European level, the European Association of Zoo and Aquaria (EAZA) elephant Taxon Advisory Group (TAG) manage the populations of African and Asian elephants to be genetically sustainable and consist of reproductively, behaviourally and physically competent individuals, which have the ability to fulfil multiple roles within a modern zoological collection [27].

The Zoological Society of London (ZSL) has been keeping elephants for over 150 years, with ZSL Whipsnade Zoo (WZ) currently holding a breeding group of Asian elephants. These individuals inhabit a custom-built exhibit within the 600-acre site and have a dedicated elephant keeping team, with support provided by on-site specialist animal management teams. Over the last decade, ZSL have made a series of coordinated investments to improve elephant welfare. This was largely through implementing new husbandry practices as well as constructing a purpose-built elephant facility to update elephant management, in line with advancing best practice. In addition to these husbandry changes, a species-specific research programme was implemented in July 2018. This programme aimed to provide data to inform animal management at WZ, as well as benefit the welfare of other elephants managed under human care in range state countries or in ex situ zoo populations.

Throughout this study, we aim to use behavioural data to understand and evaluate the development in elephant welfare through comparisons with previous published literature, with focus on the data published by Harris et al. [23]. We emphasise how information collected cannot only form a basis for evidence-based management, but also show compliance with outlined legislation for keeping elephants in a zoo environment. Additionally, we aim to outline that appropriate documentation and quantification of these advances in welfare, through targeted behavioural monitoring programmes, give collections scope to assess well-being on an individual as well as a group level. Our methods are designed to be accessible, low-cost and repeatable in other captive settings.

## 2. Materials and Methods

### 2.1. Study Subjects

**Figure 1:**
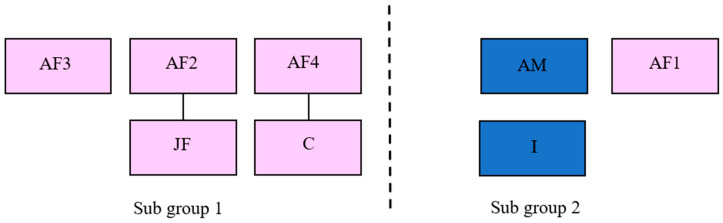
Schematic diagram representing relatedness between individuals and the composition of the two sub-groups throughout the data collection period. ‘AM’ is the sire of ‘JF’, ‘C’ and ‘I’. Both subgroups had the ability to maintain auditory, visual, and physical contact with each other through dividing fences throughout the ‘Day’ observations. Pink and blue squares refers to female and male individuals respectively, with solid lines representing a mother-calf relationship.

The subjects of this research were eight Asian elephants housed at ZSL Whipsnade Zoo, UK. The study group consisted of one adult breeding male (AM; Date Of Birth [DOB]: 10 July 1991), three post-reproductive females (AF1; DOB: 4 March 1982, AF2; DOB: 20 July 1982, AF3; DOB: 24 May 1982), one breeding adult female (AF4; DOB: 27 August 1998), one breeding juvenile female (JF; DOB: 23 July 2009), one infant male (I; DOB: 16 September 2014) and one female calf (C; DOB: 10 June 2016), all individuals were managed in a protected contact management system, since January 2018. Data from all adult animals were included in the Harris report of 2008. Age classes for this study were determined using previous published work [8]. Due to severe long-term compatibility issues between two individuals, ‘AF1′ and ‘AF2′, the eight animals were routinely housed as two subgroups to ensure the safety of all elephants (Figure 1). The dam of study subject ‘I’ is deceased and as a result ‘I’ was managed as part of Sub group 2 with ‘AM’ and ‘AF1′.

### 2.2. Data Collection

Data were collected between 19 August 2018–28 February 2019 (*n* = 139 days) with behavioural observations being divided into ‘Day’ and ‘Night’ sessions. Total observation hours were as follows: ‘Day’—213 h; ‘Night’—4656 h. Within this overall collection period, data were further classified into one of two seasonal conditions, ‘Summer’ and ‘Winter’, due to variations in housing. ‘Summer’ observations were undertaken from 19 August 2018–3 November 2018. ‘Winter’ observations were undertaken from 24 November 2018–28 February 2019. Observations were conducted when individuals were not under direct management from keepers i.e training sessions or movement between enclosures, and so had free-choice of behaviour.

The data collection schedule was randomised but balanced to ensure that all individuals and time periods were observed equally

During the winter data collection period (on 11 December 2018), ‘AF1′ was moved to another zoo upon recommendation of the EAZA Elephant TAG. Additionally, on 12 January 2019 ‘JF’ gave birth to a calf which subsequently died approximately 72 h later. For this reason, data in date range (12 January 2019–21 January 2019) was excluded from analysis as daily husbandry routine was severely disrupted during this period for all individuals due to extensive veterinary intervention.

#### 2.2.1. Day Observation Data Collection

Data were collected between 10:00–16:00 approximately, dependent upon husbandry routine, using instantaneous focal sampling (via direct, in person observations) at one-minute intervals over 30 min observation sessions [28]. Each individual was observed for at least one 30 min session per day. At each interval, the state behaviour of the focal individual was recorded using a pre-defined ethogram (Appendix A) [28]. Additionally, at each interval the ID and proximity of the focal individuals’ two nearest conspecifics were recorded in elephant body lengths (Appendix B). All occurrence sampling of pre-determined event behaviours (Appendix B) was also conducted throughout all focal observation sessions. When a social interaction occurred between the focal animal and another conspecific, the ID(s) of the participating individual(s) were recorded.

#### 2.2.2. Night Observation Data Collection

Due to restriction on camera equipment, ‘AM’ was excluded from overnight study. Data were collected between approximately 16:00–10:00, dependent upon husbandry routine, using instantaneous focal sampling (via CCTV recording) of state behaviours at 15 min intervals for the entire duration that individuals spent in overnight housing. Continuous focal sampling was used to record accurate duration of resting behaviour. Information on social resting behaviour was captured by recording the ID and proximity of the resting individuals’ two nearest conspecifics, in elephant body lengths. This data collection method allowed for multiple individuals to be observed across each ‘Night’ period. Three CCTV cameras were used for overnight recording, these were positioned to cover the entire indoor area with no blind-spots. All data collection throughout the project was carried out by the lead author, eliminating the need for inter-observer reliability assessment. Due to lack of technology and inability to individually identify all study subjects overnight, individual resting behaviour was not able to be studied in the report by Harris et al. [23]. As a result, comparisons of time spent engaging in resting behaviour will be drawn from other published literature [29,30].

#### 2.2.3. Social Behaviour

Social behaviour was recorded throughout this study as both a state and an event behaviour. When individuals were engaging in social behaviour at the one-minute sampling interval, the behaviour was classed as agonistic or affiliative social behaviour. However, to collect further detail, all occurrence sampling of key event behaviours (Appendix B) was conducted on social behaviour to record both the nature and number of bouts of social behaviour.

Index of association [28] was used to determine the extent to which each dyad, both within and between the sub groups, associated with each other throughout the study period. The index of association score ranged from 0 (no association) to 1.0 (complete association). Only scores ≥0.1 were displayed on the sociograms for clarity. Line thickness was adjusted to display strength of relationship between individuals. Proximity data were used to construct these sociograms. To gauge a true representation of the extent to which individuals were choosing to associate with each other, sociograms were constructed only using data in which individuals were either touching or within one elephant body length of each other.

### 2.3. Enclosure

#### 2.3.1. Daytime Housing

##### Day Time Housing Changed Between Seasons

‘Summer’–All individuals had access to grass paddocks throughout the summer period. Sub group 1 had access to a paddock measuring 13000 m^2^, while Sub group 2 had access to an enclosure measuring 4800 m^2^. Both paddocks had species-specific features such as wallows, sand piles and feeding sites. Sub group 1 also had access to a small pool.

‘Winter’–All individuals had access to all weather sand paddocks throughout the winter period. Sub group 1 had access to a paddock measuring 1500 m^2^. ‘AM’ and ‘I’ were housed separately in paddocks measuring 360 m^2^ and 700 m^2^ respectively. ‘AM’ and ‘I’ did not have the ability to engage in physical contact with each other throughout the winter period due to enclosure limitations; however, each individual had the opportunity to engage with Sub-group 1 through a physical barrier.

#### 2.3.2. Nighttime Housing

Overnight housing was consistent throughout seasons in purpose-built elephant barns. All individuals except ‘AM1′ were housed in an enclosure that was split into two sections ‘A’ and ‘B’ and included a deep sand substrate and multiple timed haynet feeders along with species-specific enclosure furnishings such as logs and enrichment devices. Sub group 1 were housed in ‘A’ while ‘I’ and ‘AF1′ were housed in ‘B’, with the total area of ‘A’ and ‘B’ being 700 m^2^ ‘AM’ was housed overnight in a separate bull facility of 180 m^2^. This area included rubber flooring multiple feeding sites and enrichment devices.

As this study consisted of purely observational data collection and required no changes to routine husbandry, ethical approval was not required. The project was approved following ZSL internal review (reference code ZDZ104).

### 2.4. Data Analysis

#### 2.4.1. Activity Budgets

All analyses were conducted using R statistical analysis software version 1.3.1056 [31]. To allow for comparison with data in the report by Harris et al. [23], activity budgets were calculated and presented as the proportion of time the focal individual was visible to the observer.

Each response variable was found to have a non-normal distribution (Shapiro-Wilks test: Feeding; w = 0.96, *p* ≤ 0.05, Stereotypy; w = 0.54, *p* ≤ 0.05, Resting; w = 0.89, *p* ≤ 0.05, Anticipatory; w = 0.42, *p* ≤ 0.05). As appropriate transformations could not be applied, non-parametric statistical tests were used for these variables. Resting was also calculated in minutes, these data were tested separately for normality (Shapiro-Wilks test: w = 0.98, *p* = 0.98) and were found to be normally distributed. As a result, parametric statistical tests were used when analysing these data. To allow for accurate comparison to published literature, resting duration data were only used between time periods 19:00–08:00 in analysis.

#### 2.4.2. Data Analysis–Sociograms

Sociograms were made using NetDraw Network Visualisation Software 2.172 package on UCINET 6.709 [32], using information obtained by calculating an index of association [28] for each dyad within and between the sub groups.

## 3. Results

### 3.1. Feeding

**Figure 2:**
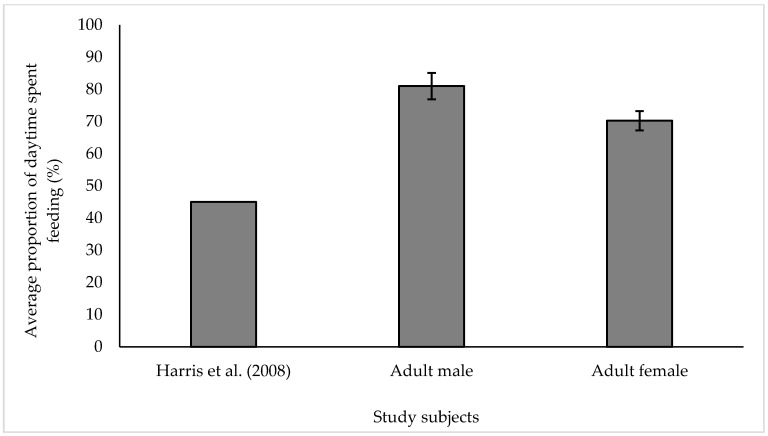
Average proportion of time spent feeding throughout ‘Day’ observations for the adult male and all adult females in study group. Values from published literature taken from Harris et al. [23]. All values displayed as mean ± s.e.

All types of feeding behaviour described in Appendix A were grouped together for the purpose of analysis and presentation of results. All food-stuff was presented in a way to mimic natural foraging behaviour and extend foraging time, all hay was presented in elevated hay nets while browse was either hung up on winches or presented throughout the enclosure at ground level. Throughout the summer, individuals were housed on grass paddocks which further encouraged natural foraging behaviour in the form of grazing for grass. Individuals were encouraged to use the whole exhibit through placement of browse and hay nets at opposite ends of the enclosure. To allow for direct comparison with Harris et al. [23], younger individuals were not included in Figure 2 or in statistical analysis.

Average proportion of daytime spent engaging in feeding behaviour for the adult male and adult females was significantly higher than the value of 45% published by Harris et al. [23] (Wilcoxon signed rank test: AM; w = 496, *p* < 0.001, Adult females, w = 2346, *p* < 0.001). The averages from this study show that the WZ adult male spent 80.99% (±4.12 s.e) of his day engaging in feeding behaviour, while our adult females spent 70.23% (±3.03 s.e) of their day feeding (Figure 2). Average time spent feeding throughout the day for the juvenile female (JF) was 77.31% ± 4.05 s.e; infant (I) 50.71% ± 4.36 s.e and calf (C) 49.07% ± 4.07 s.e.

### 3.2. Stereotypic Behaviour

All stereotypic behaviours (Appendix A) observed were grouped together for purpose of analysis and presented here as such. To make data directly comparable to those presented by Harris et al. [23] stereotypies were calculated as proportion of engagement over a 24 h period. Due to enclosure limitations with CCTV equipment, ‘AM’ could not be observed over a 24 h period, thus this individual was excluded from the graph below.

**Figure 3:**
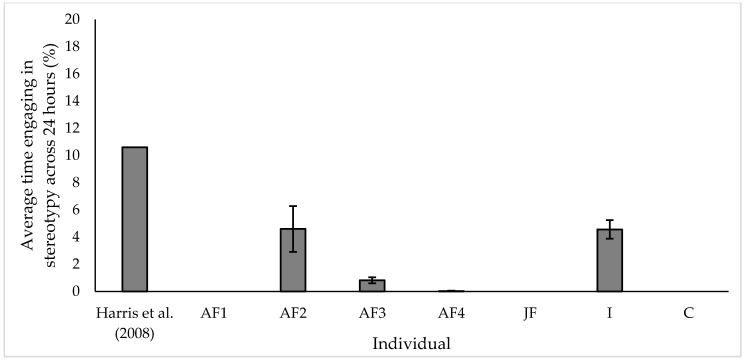
Average proportion of time spent engaging in stereotypic behaviour compared to historic figures published in Harris et al. [23]. All values displayed as mean ± s.e.

Of the eight individuals within the study group, 3 individuals were not observed engaging in any form of stereotypic behaviour (Figure 3). Of the individuals that did engage in stereotypic behaviour, all displayed significantly lower levels of stereotypy than the average for UK Asian elephants in 2008 as stated by Harris et al. [23] (Figure 3: Wilcox signed rank test; AM, w = 2278, *p* < 0.001; AF2, w = 496, *p* < 0.001; AF3, w = 2701, *p* < 0.001; AF4, w = 1485, *p* < 0.001; I, w = 496, *p* < 0.001). The most common stereotypic behaviour observed in our individuals was pacing, accounting for 76.6% of all stereotypic behaviour observed, followed by swaying (15.9%) and then weaving (7.5%).

### 3.3. Resting

**Figure 4:**
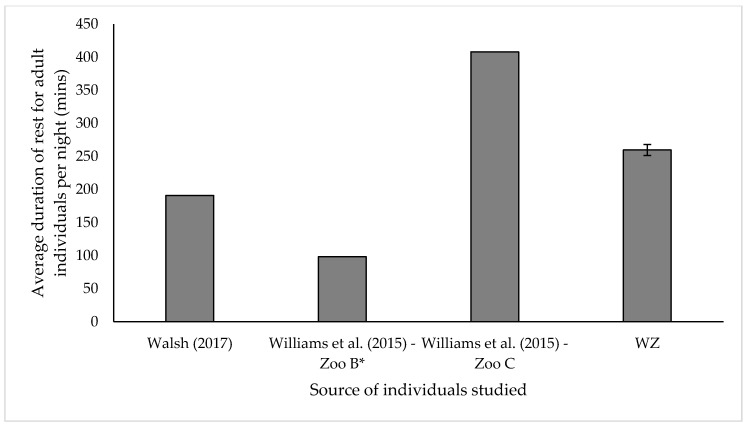
Comparison of the average time spent resting (min) per night between adult individuals at WZ and those published by Walsh [29] and by Williams et al. [30]. All values displayed as mean ± s.e. * Values from Williams et al. [30] Zoo B only included duration of lying rest.

Resting data were calculated as both average proportion of time over 24 h (%) and as duration per night (mins). For the purposes of data presentation and analysis, both lying and standing rest were grouped (Table 1 and Figure 4). All individuals had access to deep sand substrate only throughout the course of data collection.

Upon comparison with published data from another European institution [29] and from other UK Zoos [30], the duration of time spent resting in minutes, did not significantly differ between WZ adults and Walsh [29] adults (Figure 4, One sample *t*-test: Walsh [29], *t* = 55, *p* = 0.13). However, a significant difference was found in the average duration of rest per night for adult individuals sampled by Williams et al., 2015 in both Zoos B and C when compared to adult individuals at WZ (Figure 4, One sample *t*-test: Williams et al. [30] Zoo B, *t* = 8.6, *p* ≤ 0.05; Williams et al., (2015) Zoo C, *t* = 5.5, *p* = 0.01) ‘Zoo A’ from Williams et al. [30] was excluded from data collection, as individuals did not have access to sand substrate, a substrate provided for all other study individuals.

**Figure 5:**
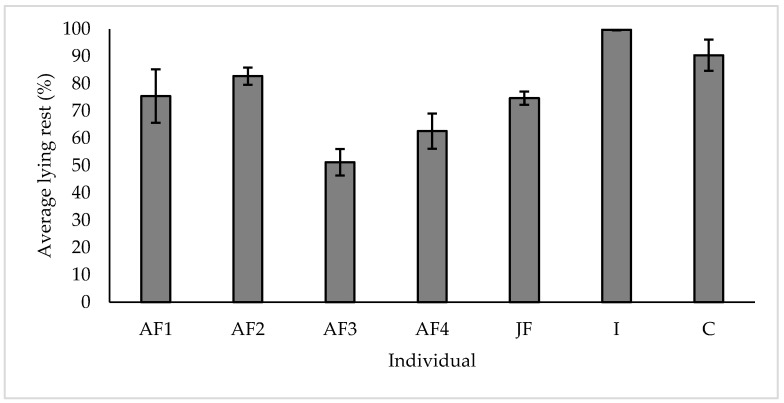
Average proportion of resting time spent in lying rest for each subject over 24 h. All values displayed as mean ± s.e.

When evaluating the average proportion of time spent resting over 24 h (%), all individuals at WZ studied spent more than 50% of their time engaging in lying rest behaviour, with the youngest group members ‘I’ and ‘C’ spending the most time engaging in lying rest (99.69% ± 0.15 s.e and 90.39% ± 5.72 s.e respectively) (Figure 5). 

### 3.4. Social Behaviour 

**Figure 6:**
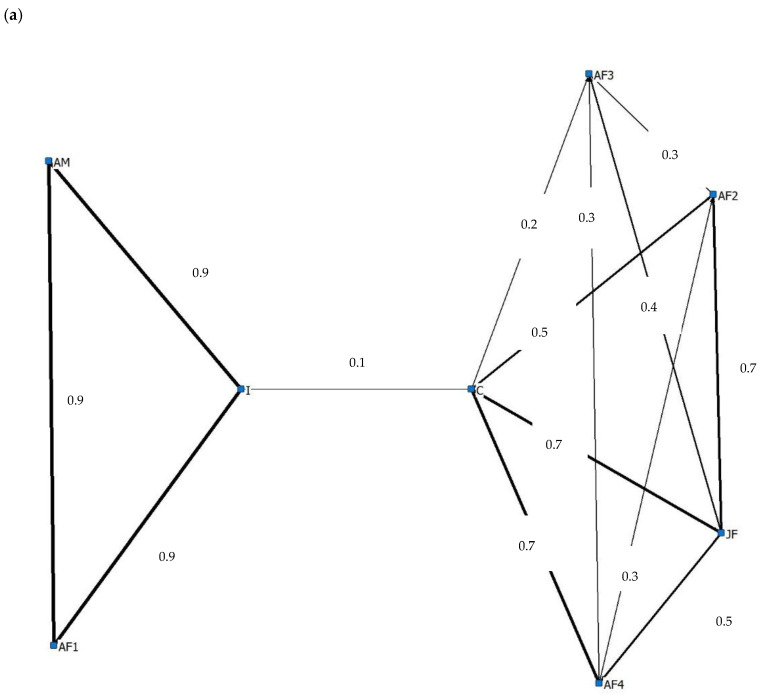

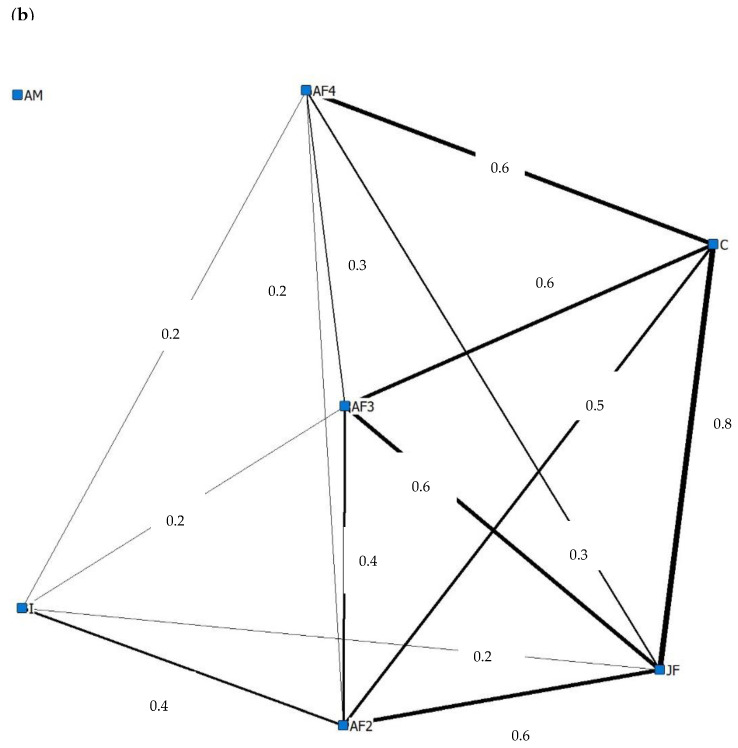
A sociogram highlighting index of association scores ≥0.1 between members of the study group using social proximity data collected throughout the ‘Day’ observation period while individuals were housed in summer (**a**) and winter (**b**) accommodation. Interactions involving ‘I’ in Figure 6b were through a fence, due to enclosure design.

Due to seasonal changes in housing resulting in a difference in enclosure size (Methods 2.3), sociograms were constructed for both the ‘Summer’ housing (Figure 6a) and ‘Winter’ (Figure 6b) housing observation periods.

The strongest index of association scores in sub-group 1 were observed between ‘JF’ and ‘C’ (0.7 and 0.8 summer and winter respectively), ‘AF4′ and ‘C’ (0.7 and 0.6 summer and winter respectively) and ‘AF2′ and ‘JF’ (0.7 and 0.6 summer and winter respectively). Throughout the summer period (Figure 6a), Sub group 2 had high index of association scores between all dyads (0.9). Throughout the winter period, ‘I’ chose to associate with ‘AF2′, ‘AF3′, ‘AF4′ and ‘JF’. ‘AM’ chose to associate less frequently with members of Sub group 1 leading to the removal of this individual from the core sociogram (Figure 6b).

**Figure 7:**
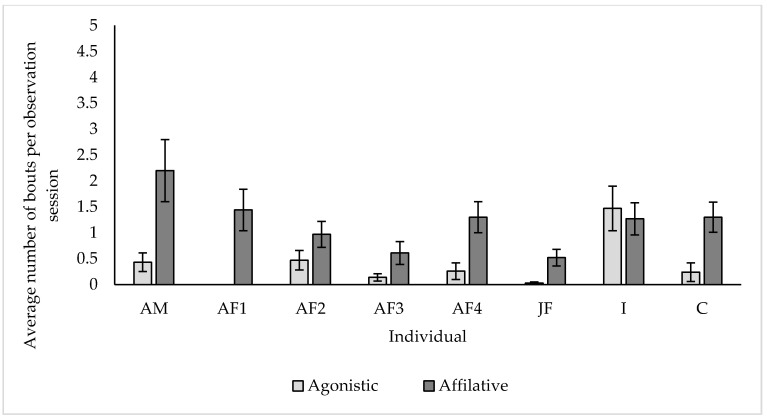
Average number of bouts of affiliative and agonistic social interactions initiated per individual, per observation session. All values displayed as mean ± s.e.

Affiliative social behaviour was recorded as a state behaviour for all eight of our study individuals. The highest average proportion of engagement in affiliative social behaviour observed was from ‘C’ (8.71% ± 2.1 s.e) and the lowest from ‘AF1′ (0.63% ± 0.54 s.e). Agonistic social behaviour was not observed as a state behaviour throughout our study period. Both affiliative and agonistic social interactions were recorded as event behaviours for seven of the eight individuals throughout the period of observations (Figure 7).

Per observation session on average, ‘AM’, ‘AF1′, ‘AF3′, ‘AF4′, ‘JF’ and ‘C’ all initiated more bouts of affiliative than agonistic social behaviour throughout our study period On average, study subject I engaged in the most agonistic bouts per session (1.47 ± 0.43 s.e) while ‘AM’ engaged in the most affiliative bouts per session (2.2 ± 0.6 s.e).

### 3.5. Anticipatory Behaviour

**Figure 8:**
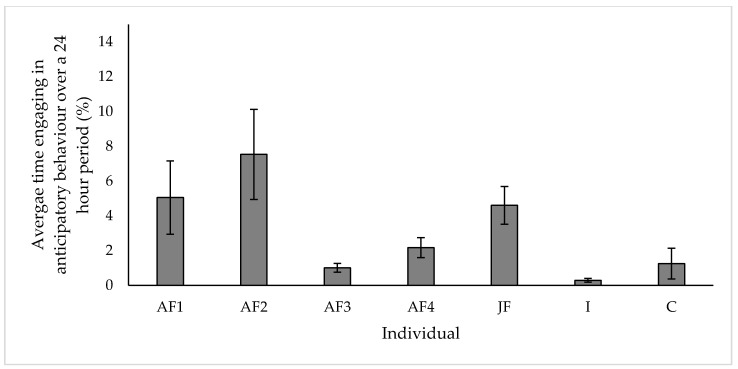
Average proportion of time spent by each study individual engaging in anticipatory behaviour. All values displayed as mean ± s.e.

Anticipatory behaviour formed a proportion of the activity budget for all individuals studied, with ‘AF2′ showing the highest proportion of anticipatory behaviour (Figure 8; 7.53% ± 2.59 s.e) and ‘I’ showing the lowest (Figure 8; 0.29% ± 0.11 s.e). This behaviour was almost exclusively observed between 07:00–09:00 (51.8% of all anticipatory behaviour observed) and 14:00–15:00 (44.7% of all anticipatory behaviour observed), before individuals were moved into either daytime or overnight housing.

## 4. Discussion

### 4.1. Feeding

Asian elephants are known as generalist herbivores, consuming a variety of plant material including bark, twigs, fruit and leaves [33] with estimates of daily dry matter intake for a wild adult elephant to be around 1–1.5% of their body mass [34]. Elephants have a low digestive efficiency and are designed to eat large quantities of nutrient poor fibrous material, which passes quickly through the gastrointestinal tract [35,36].

Average values for proportion of daytime feeding behaviour collected throughout our study (Adult male 80.9% ± 4.1 s.e; Adult female 70.2% ± 3.0 s.e) are significantly higher than those stated by Harris et al. [23], who stated that at the time of data collection, feeding represented 45% of a UK captive elephants activity budget. Studies focusing on wild elephant ecology show that individuals spend up to 67% of their time feeding [33] and that elephants are extremely opportunistic in their food choice, adjusting their movements, food choices and subsequent behaviour-based upon mineral need [37].

Increased time spent feeding compared to the UK average stated by Harris et al. [23] may be due to the seasonal provision of grass at WZ. These findings are consistent with studies on wild individuals, Sukumar [33] suggested that wild Asian elephants will increase their time spent feeding on grasses when they become seasonally available. Grass is highlighted as an important resource for elephants in section 8.8.34 of the SSSMZP [19] and required in the BIAZA Best Practice Guidelines for these species [38], to maximise and extend foraging time for zoo individuals without significantly increasing the quantity of food consumed and putting the species at increased risk of obesity. Since the publication of data in Harris et al. [23] elephant holders have made a concerted effort to increase foraging time and promote natural foraging behaviour, through targeted enrichment, increased provision of browse, timed feeders, and the use of raised hay nets [39,40,41]. To meet the nutritional needs of these species and reduce the risk and incidence of obesity within zoo elephants, further work is required to develop low quality palatable forages that are better suited to the nutrient poor high fibre needs of the species, rather than UK grass hays which are designed primarily for the nutritional needs of domestic farm animals.

### 4.2. Stereotypy

Stereotypic behaviours are one of the most common behavioural measures used to indicate zoo animal welfare status [40]. In elephants, the most common stereotypies include pacing, weaving, and swaying [41], with Harris et al. [23] reporting that swaying was the most common stereotypy in the UK elephant population. Swaying was recorded by Harris et al. [23] to form 67% and 78% of all daytime and nighttime stereotypies observed respectively. Harris et al. [23] reported locomotor stereotypy to be the next most common stereotypic behaviour (27% and 11% of day and nighttime stereotypy respectively). Of stereotypies reported in our study group, individuals engaged the most in locomotor stereotypy (76.6% of all stereotypy observed), followed by swaying (15.9% of all stereotypy observed). Additionally, a questionnaire developed by Hapeslagh et al. [42] which surveyed 87 elephants across 12 European zoos, also concluded that locomotor stereotypies were the two of the most common, with weaving and pacing forming 37.9% and 17.2% of all stereotypies observed.

Individuals engage in stereotypic behaviour for several reasons, with both biotic and abiotic factors linked to the frequency of occurrence such as enclosure size [43], husbandry routine [42] and health status [44]. Engagement in stereotypic behaviour can be a product of a previous poor welfare experience, with work suggesting that these forms of stereotypy may persist, despite positive changes to the current environment [45,46]. Engagement in stereotypic behaviour from our study subjects was found to be significantly lower than those published by Harris et al. [23]. A reduction in levels of stereotypy has been suggested to indicate an improvement in welfare state for many captive species [47,48], and linked to positive advances made in husbandry practices [49] and animals feeling comfortable in their environment [50].

Furthermore, our results highlight the importance of evaluating welfare on an individual level in this species, with the type of stereotypy and proportion of time spent engaging in abnormal repetitive behaviour varying greatly between study subjects. Each individual within our study group, particularly the older adult females, had a unique and sometimes complex life history, with experienced multiple management systems, facilities, and husbandry practices. Therefore, development of husbandry practices and investment in facilities may reduce levels of stereotypy for these individuals but ultimately they never fully remove this less desirable behaviour from their activity budget. Continual improvement in evidence-based husbandry and appropriate intervention will prevent or at least significantly reduce the likelihood of younger animals developing stereotypies into the future.

### 4.3. Resting

While observational data on resting behaviour in wild Asian elephants is scarce, engagement in lying rest is considered important for this species and is used as an indicator for positive welfare in zoo individuals [51]. Absence of lying rest behaviour could be linked to environmental factors such as substrate provision [30] or physiological health conditions such as degenerative joint disease or impaired musculoskeletal strength [52] especially in geriatric individuals. At the time of data collection, WZ elephants were all of a good health status, with no diagnosed cases or signs of disease or physical impairment. Our results show that all study subjects engaged in nightly lying rest and all individuals did so for more than 50% of their overall resting period. Our results support findings of another study which highlighted that duration of rest decreased with age [53], with our calf and infant study subjects engaged in the highest proportion of both lying and overall rest. WZ invested in providing a substrate of deep sand throughout the indoor facility, housing all individuals except ‘AM’. The provision of appropriate substrate and enclosure furnishings were suggested to encourage safe, positive lying rest in elephants, with deep sand recommended as the most suitable substrate for elephants [54]. Due to research into the effect of substrate on elephant health [30,55], many UK elephant holding zoos have transitioned to more species-appropriate substrates, since the report by Harris et al. [23]. Additionally, appropriate social groupings overnight are found to be related to positive lying rest, with individuals with longer resting bouts when a conspecific was within two elephant body lengths than when conspecifics were not [56]. The duration of rest behaviour for our study subjects was recorded and was found to be consistent with data published from another large elephant holder within Europe [29]. These findings highlight the importance of collaboration and dissemination of research across the zoo community, particularly in the UK, in order to promote a holistic, institution-wide approach to evaluating welfare.

### 4.4. Social Behaviour

Elephants are a highly social species [57], with wild herds formed of multiple matrilines and led by a matriarch. Individuals live in a fission fusion society, with the integration or dispersal of group members dependent on multiple factors such as resource availability [58] or reproductive status [59]. It is now recommended that elephant holders should aim to replicate the natural social structure of wild elephant herds within a zoo setting to ensure optimum welfare [59,60]. Our study group is made of both related and unrelated members and we have used sociograms to demonstrate the extent to which individuals chose to associate with another throughout our study. Our sociograms demonstrate that individuals within the study group are exercising a level of choice and control over their association with other group members, with individuals within and between each sub group actively choosing to spend time in close proximity. Throughout the summer data collection period, the strongest associations were observed between all members of Sub group 1, the mother-calf dyads in Sub group 2 and half-sister dyad between ‘JF’ and ‘C’. Our findings are consistent with studies on both wild and zoo individuals, highlighting the importance and strength of the mother—Calf relationship [57,61,62]. The strong association we observed between ‘JF’ and ‘C’ further highlights the importance and benefits of keeping this species in related social groupings. However, it is also important to highlight that ‘AF3′, an unrelated female in this group has formed associations with all other members in her sub group. This level of association was maintained throughout both summer and winter housing periods, showing that while not related, this individual can both form and maintain cohesive social associations with group members. Despite these cohesive social associations, ‘AF3′ engaged in the lowest rates of affiliative behaviour with other group members. Suggesting that while unrelated individuals can co-exist within a herd setting, the role they play is not as important as one of a matrilineal relationship. This work revealed that six of the eight study individuals engaged in significantly more affiliative than aggressive social interactions throughout our study period. Opportunities for engagement in affiliative social interactions have been suggested to be very important in zoo individuals [63], particularly for complex and social species such as elephants [11]. Elephants use social encounters to both form and maintain social relationships, with group cohesion and stability used as an indicator of welfare experience in captivity [64]. Updated legislation recognises the importance of compatibility, stating that elephant groups should consist of at least four compatible females over 2 years old [19].

Additionally, our results highlight the important, positive influence of integrating males of different ages into socially compatible groups where possible. The strong association index observed between ‘AM’ and ‘I’ in the ‘Summer’ was not present throughout ‘Winter’ observation sessions as enclosure limitations prevented ‘AM’ socialising with ‘I’ throughout the winter. In the wild, male elephants are reported to socialise with conspecifics, forming batchelor groups with other males [65,66,67,68] and developing social relationships with females that may be maintained throughout their lives [67]. Hartley et al. [67] highlight the issues surrounding social management of male elephants in zoos and the importance of adopting new approaches to management in the future. Our data is further evidence that investment is needed in facilities to allow the free socialisation of males in a fission fusion system, during all weathers.

### 4.5. Anticipatory Behaviour

Anticipatory behaviour, often described as the actions taken to prepare for an upcoming event [68], has been linked to zoo animals living in highly predictable environments with specific husbandry routines [69]. This behaviour has been recorded in multiple captive species [70]. However, the interpretation of anticipatory behaviour in relation to animal welfare experience must be done with caution [71]. Associated with the release of dopamine, anticipatory behaviours were used as an indicator of positive welfare in some species [72]. However, Watters [71] outlined that the development of anticipatory behaviours escalated to a point that they became detrimental to a naturalistic activity budget. Engagement of anticipatory over more natural species-specific behaviour is a cause for welfare concern. Anticipatory behaviour in our study subjects was almost exclusively observed towards the end of ‘Day’ and ‘Night’ observations before the event of moving into a new enclosure or starting their daily husbandry routine. Engagement in anticipatory behaviour must be closely monitored through further research to assess how this behaviour influences the activity budget of the study group. Further mitigations must be investigated, especially during the ‘high risk’ times of end of ‘Day’ and ‘Night’ when significant events occur, resulting in this observed anticipatory behaviour. Dependency on these events must be minimised as much as possible, for example through enabling 24 h indoor/outdoor access (weather permitting) and conducting the daily husbandry routine and training on a flexible, opportunistic schedule, to lessen the dependency on these critical time points.

## 5. Conclusions

Throughout this study, we emphasised the value of implementing a species-specific research programme in adopting an evidence-based approach to husbandry and management. Data collected through the initial period of this long-term monitoring programme, shows that since the publication of a report on UK elephants by Harris et al. [23] there were positive advances in elephant welfare at ZSL Whipsnade Zoo. We documented that individuals in our study group engaged in significantly less stereotypic behaviour than reported by Harris et al. [23] that individuals spent longer engaging in natural feeding behaviour and that these individuals form and maintained strong social associations with one another across seasons. We outlined that investment and advancement in facilities enabled us to observe our study group overnight on an individual level, allowing detailed information to be documented on resting behaviour, with a particular focus on the engagement in lying rest. When compared with published literature from another large European elephant collection [31], we found statistical concordance between duration of rest for all comparative age classes, showing the importance of a collaborative approach to assessing welfare between institutions. We acknowledge that within our collection there is still work to be done to achieve what is currently considered optimum elephant welfare and that programmes must continually evolve in line with ever advancing best practice. Examples of suggested improvements by the authors include providing all individuals with 24 h access to outdoor enclosures, increased grazing and access to browse particularly over the winter period and an increased provision for male socialisation within future enclosure design. Long-term management plans are currently in place to work to achieve these goals over the coming years, along with continued effort to develop a cohesive matriline structure within the study group. This evidence-based assessment signifies a key step in the evolution of modern elephant management, by using both positive and negative indicators of wellbeing and an individual approach to the assessment of welfare. We also acknowledge areas for future development within our research programme, such as the current lack of focus on overnight male behaviour, identifying a need for further investment in camera resource to gain a 24 h insight into bull welfare within our collection.

## Figures and Tables

**Table 1:** Average proportion of time spent resting (%) over 24 h and the average duration of resting (minutes) per night by each study subject at WZ.

Individual	Average Proportion of Time Spent Resting per Night (% ± s.e)	Average Duration Spent Resting per Night (Minutes ± s.e)
AF1	24.9 (±2.6)	243.8 (±12.6)
AF2	26 (±1.5)	296.7 (±18.3)
AF3	24.2 (±2.8)	246.1 (±15.8)
AF4	21 (±1.2)	246.8 (±13.5)
JF	28.6 (±0.9)	343.9 (±10.5)
I	31.5 (±0.8)	338.3 (±10.5)
C	29.8 (±2)	343.2 (±16.7)

## Appendix A

**Table A1:** Ethogram outlining state behaviour definitions—Behaviours were recorded at 1 min intervals throughout the observation session. Adapted from Asher et al. [51] and Williams et al. [19].

Behaviour Category	Behaviour	Description
Anticipatory	Anticipatory	Individual stands in a stationary, quadrupedal position at a fixed point within the exhibit. Attention is often fixed at this point, with head orientated towards it and no other discernible behaviours taking place.
Natural self-maintenance	Digging	Individual using foot to displace substrate.
Natural self-maintenance	Dust bathing	Individual uses trunk to pick up sand, dust or other substrate and throw it over the body.
Natural self-maintenance	Grooming self	Individual is scratching or rubbing itself using trunk or against an enclosure furnishing.
Natural self-maintenance	Wallowing	Individual is interacting with the mud wallows, rolling their body in the muddy water.
Natural self-maintenance	Water bathing	Individual using trunk to suck up water then spray and throw over the body.
Natural exploratory	Enrichment manipulation	Interaction with pieces of enrichment provided by the keepers as part of their enrichment plan.
Natural exploratory	Object manipulation	Interaction with other objects within the enclosure other than enrichment.
Feeding	Feeding	Active consumption or manipulation of feed items or ingestion of water. Includes foraging for grass, browse and hay.
Keeper interaction	Keeper interaction	Any interaction between the individual and members of the animal keeping team.
Stereotypy	Stereotypy	Individual is displaying an abnormal, repetitive behaviour which does not have a clear purpose or outcome. This can include: Linear pacing—Individual is walking forwards then backwards in a repetitive fashion. Pacing—Individual is locomoting in a repetitive fashion in a set pattern or route which serves no specific function. Swaying—Individual transfers weight onto alternate limbs in a repetitive pattern while in a fixed position, direction of travel can be from side to side or front to back. No head movement. Weaving—Individual transfers weight onto alternate limbs while simultaneously moving the head from side to side.
Locomotion	Walk	Individual moving across the enclosure to get to another specific location at a walking pace. Only one foot is removed from the ground at any one time.
Locomotion	Run	Individual moving across the enclosure to get to another specific location at a running pace, more than one foot in removed from the ground at any one time.
Resting	Lying rest	Individual is relaxed in lateral recumbence.
Resting	Standing rest	Individual is upright and stationary with 3 or 4 feet on the ground. Individual is relaxed with no other behaviour being displayed.
Affiliative social	Affiliative social behaviour *	Any social behaviour which maintains or strengthens positive social relationships within the group.
Agonistic social	Agonistic social behaviour *	Individuals interacting in a way which may disrupt or weaken social bonds within the group.

* Nature and type of social interaction explained in more detail in ‘event behaviour sampling categories’.

## Appendix B

**Table A2:** Ethogram outlining event behaviour definitions—Behaviours were recorded on an all occurrence basis throughout the observation session. Adapted from Asher et al. [51] and Williams et al. [19].

Behaviour Category	Behaviour	Description
Affiliative social	Trunk hold	Individual has its trunk intertwined with conspecific’s trunk in a non-aggressive manner.
Affiliative social	Trunk touch	Individual places trunk in a non-aggressive manner on the body, mouth or genital area of a conspecific.
Affiliative social	Body rub or nudge	Individuals have gentle physical contact with one another, head-head, head-body, body-body.
Affiliative social	Play	A positive interaction which could include sparring, wrestling, mounting, chasing, and rolling on another elephant in a playful manner.
Affiliative social	Leaning	Elephant gently leaning on a conspecific with their head, side or rump.
Affiliative social	Urine and faecal inspection	Individual inspects the urine or faeces of another elephant.
Affiliative social	Nursing	Mother stands for calf to suckle.
Agonistic social	Kick	Individual strikes out and hits another elephant with their foot in an aggressive manner.
Agonistic social	Trunk slap	Hitting a conspecific with trunk, head orientated towards another elephant violently swinging trunk in an aggressive display.
Agonistic social	Charge	Individual moving at a fast pace towards conspecific for more than three steps, head held high with some contact made.
Agonistic social	Mock charge	Individual moves at a fast pace towards conspecific for more than three steps, contact does not occur.
Agonistic social	Chase	Follows on from ‘charge’ behaviour leading to the pursuit of an individual.
Agonistic social	Push	Elephant forces or pushes body of another elephant, resulting in elephant that is pushed moving at least two steps.
Agonistic social	Stand off	Two elephants standing facing in opposite directions, with foreheads pushing against each other.
Agonistic social	Lunge	Individual thrusts their body towards a conspecific in an aggressive manner taking less than three steps. If more steps taken then reclassify as ‘Charge/Mock charge’.
Agonistic social	Tusking	Individual pokes or jabs a conspecific with their tusk.
Agonistic social	Tail pulling	Individual pulls a conspecifics tail with trunk
Agonistic social	Contact displacement	Movement of an individual resulting in conspecific leaving its location (within 10s) caused by physical contact between individuals such as push or nudge.
Agonistic social	Non-contact displacement	Movement of one elephant towards the other, resulting in conspecific leaving its location (within 10s) no physical contact occurs between elephants.
Agonistic social	Aggressive display	Facing a conspecific in an aggressive posture, head bobbing up and down or side to side, ears wide and flapping.
Agonistic social	Biting	Individual bites a conspecific’s body, trunk or tail. Physical contact occurs between the mouth of the initiator and the body part of the recipient.
Agonistic social	Trunk dominance	Focal individual places their trunk over the top of conspecific, mouths usually close together. Individual actively tries to place trunk in a higher position than conspecific to assert dominance.
Agonistic social	Food stealing	Individual takes food away from a conspecific using its trunk or another part of the body.
Excretion	Urination	Elimination of urine from the body.
Excretion	Defecation	Elimination of faeces from the body.
Vocalisation	Trumpet call	Individual emits a loud alarm call.
Vocalisation	Rumble	Individual emits a low frequency rumble call.

### Proximity Categories

While conducting observations on the focal individual, at each interval please note who their two nearest neighbours are and the approximate distance (in elephant body lengths) the two individuals are away from the focal animal.

0— Individuals are touching but not engaging in a social interaction
1— Individual is one elephant body length away from the focal individual
2— Individual is two elephant body lengths away from the focal individual
3— Individual is three or more elephant body lengths away from the focal individual.
